# The Role of Emotion-Related Abilities in the Quality of Life of Breast Cancer Survivors: A Systematic Review

**DOI:** 10.3390/ijerph191912704

**Published:** 2022-10-04

**Authors:** Ilaria Durosini, Stefano Triberti, Lucrezia Savioni, Valeria Sebri, Gabriella Pravettoni

**Affiliations:** 1 Applied Research Division for Cognitive and Psychological Science, IEO, European Institute of Oncology IRCCS, 20141 Milan, Italy; 2 Department of Oncology and Hemato-Oncology, University of Milan, 20123 Milan, Italy

**Keywords:** breast cancer, coping, emotional intelligence, emotions, quality of life, cancer survivorship, mood repair

## Abstract

Breast cancer survivors have to deal with notable challenges even after successful treatment, such as body image issues, depression and anxiety, the stress related to changes in lifestyle, and the continual challenges inherent to health management. The literature suggests that emotional abilities, such as emotional intelligence, emotion management, mood repair, and coping play a fundamental role in such challenges. We performed a systematic review to systematize the evidence available on the role of emotional abilities in quality of life and health management in breast cancer survivors. The search was performed on three scientific databases (Pubmed, Scopus, and PsycINFO) and, after applying exclusion criteria, yielded 33 studies, mainly of a cross-sectional nature. The results clearly support the hypothesis that emotional abilities play multiple important roles in breast cancer survivors’ quality of life. Specifically, the review highlighted that coping/emotional management plays multiple roles in breast cancer survivors’ well-being and health management, affecting vitality and general adjustment to cancer positivity and promoting benefit findings related to the cancer experience; however, rare negative results exist in the literature. This review highlights the relevance of emotional abilities to promoting quality of life in breast cancer survivors. Future review efforts may explore other breast cancer survivors’ emotional abilities, aiming at assessing available instruments and proposing tailored psychological interventions.

## 1. Introduction

Breast cancer is the most common and curable type of cancer in women [1]. Last decades’ improvements in radiotherapy, chemotherapy, surgery, as well as in the precision medicine approach notably improved the number of breast cancer survivors. Breast cancer survivors are people who are still alive five years after diagnosis and completed the oncological treatments [2,3,4]. In this sense, cancer survivors have rapidly increased due to the increase in the proportion of the elderly, the growing rates of detection and incidence, and improvement in survival [5,6]. For example, more than 53,000 women annually receive a breast cancer diagnosis in Italy alone [7]. However, successful cancer treatment does not mean that survivors have reached a satisfactory level of health and well-being. On the contrary, breast cancer survivorship is associated with several physical and psychological issues possibly lasting for one’s life. 

Firstly, cancer treatments bring along issues that negatively affect cancer survivors’ body image, chronic pain, and quality of life [8]. Undesirable appearance-related side effects, such as visible scarring, hair loss, skin discoloration, muscle weakness, loss or deformities in the breasts, and weight fluctuation, affect physical appearance so that the survivors develop intensive negative feelings, such as anxiety and depression [9,10,11,12]. Moreover, sexual dysfunctions frequently lead to dissatisfaction in intimate relationships [12,13,14,15]. Secondly, because of the need for continuous care and monitoring, breast cancer survivors have to make and maintain important changes in their lifestyles and life plans (e.g., if and how to return to work or usual activities), which can be associated with stress and persistent negative emotions [16]. Lastly, women with a history of tumor typically experience fear of recurrence [17,18,19,20], namely the persistent fear that cancer may return, which could generate pathological anxiety, depressive episodes, and notable fluctuations in survivors’ motivation to continue managing their own health. It is well-established in the scientific literature that emotions play a major role in all of these processes. Emotions are the cognitive and physiological processes that allow people to understand the relevance of stimuli and events in the external environment for their present life and objectives [21]; the arousal or physiological activation generated by the perception of the stimulus is elaborated by an appraisal process that allows the individual to identify the behavior to respond to the stimuli (e.g., approaching a pleasant stimulus or avoiding a dangerous one). However, people differ in their ability to deal with their emotions and consequently in adopting optimal reactions to avoid any dysfunctional effects of environmental stimuli and emotions themselves [22,23]. For this reason, it is important to develop tools and resources to assess and possibly improve people’s ability to recognize and manage emotions, especially within delicate contexts such as chronic health management. Secondarily, the experience of emotions directly influences the outcomes of decision-making; emotions affect attention, memory, and notably contribute to the generation of meaning [24,25]. A breast cancer survivor who would be proficient in recognition of her own and others’ emotions would also be more able to make optimal decisions regarding her care, and reduce the influence of cognitive biases typical of chronic disease management [26] as well as make good use of social support resources. 

Given the prominent role of emotions in breast cancer survivors’ quality of life and health management, it is important to consider the individual abilities to understand and manage emotions so that they do not constitute an obstacle to health and well-being [27]. Psychological research has identified several abilities, sometimes different, partially overlapping constructs that pertain to the effective management of emotions. Emotional intelligence, functional coping, emotional management, and mood repair are all concepts that belong to psychological science’s theoretical background and pertain to the abilities to recognize, manage, and make good use of emotions to achieve health and well-being. 

Emotional intelligence could be defined as a type of social intelligence that involves the ability to perceive, monitor, and express one’s and others’ emotions, discriminate among them, and use such information to guide and manage one’s thinking and actions voluntarily [28,29]. While this popular concept has yielded an impressive number of studies in the last decades, it is still an object of controversy [30]. Specifically, psychologists debate whether emotional intelligence should be considered a collection of cognitive capacities, to be measured by performance tasks similar to those used to measure “cognitive” intelligence, or a complexity of convictions about one’s abilities regarding emotions, plausibly rooted in successful mastery of emotion-related tasks, ultimately similar to a specific type of self-efficacy or a personality trait. While developing emotional intelligence measurement tools based on performance tasks is difficult (e.g., how to determine the “right emotions” to feel within a given scenario?), the trait-like conception of emotional intelligence has allowed researchers to develop some self-report measures such as the TEIQUE and the BEIS. These scales demonstrated good reliability as well as the capacity to predict quality of life and health management outcomes [31]. 

Across emotion studies, coping and emotional management are broad terms that refer to the cognitive strategies an individual may use to manage emotions, especially when trying to avoid excessive emotional activation negatively influencing daily life. Most theoretical models on coping distinguish between multiple strategies. In general, the research shows that dysfunctional strategies relate to disengagement, avoidance, or the suppression of the emotion; those strategies tend to be effective in the short term, but they are scarcely related to long-term achievements and positive behavioral change. On the contrary, mature strategies involve re-appraisal and problem solving focused on the emotional stimulus, and are positively associated with several aspects of well-being and negatively related to distress or other negative states.

While emotions are usually conceptualized as the response to specific internal or external stimuli, mood can be defined as a diffuse, nonspecific affective state which lasts relatively long periods (e.g., days) [32,33,34]; a person in a “bad mood” may be unsure about the exact reason for his or her emotional state, yet may feel more or less impaired in some tasks (e.g., putting low effort in everyday activities; engaging in rumination and negative thoughts). In this sense, mood repair refers to the ability to modify one’s mood, for example by voluntarily recovering positive memories or engaging in pleasant activities [35]. 

The research on emotional abilities in cancer survivors can be traced back to the first decade of 2000, with a strong focus on coping/emotion regulation. A recent meta-analysis [36] on emotion regulation and its effects on psychological distress in cancer survivors (not only breast) found studies equally distributed across the globe (Europe: five; Americas: four; and Asia: six). However, the authors also reported that high variability among the reviewed studies (e.g., type of cancer and country), also considering their relatively small number, made it difficult to explain some inconsistent results (i.e., positive, negative or inexistent association between emotion regulation strategies and distress). Furthermore, only studies on emotional suppression were included in the meta-analysis, possibly implying that the literature in its entirety was not ready for meta-analytic efforts. In any case, it is still paramount to identify the role of emotional abilities in psychological processes relevant to the quality of life and health management, not only psychological distress; therefore, we planned the present systematic review limiting the focus to breast cancer (along with its specific psychological issues described above) but extending it regarding all emotional abilities and variables relevant to breast cancer survivors’ quality of life and well-being. 

On these bases, we conducted this systematic review to identify the role of emotion-related abilities (emotion regulation/coping, emotional intelligence, and mood regulation) in breast cancer survivors’ overall quality of life. Given the complexity of the topic, we adopted a broad approach by systematically searching for studies that assess the multiple constructs that fall under the aegis of emotion-related abilities.

## 2. Methods

**Figure 1:**
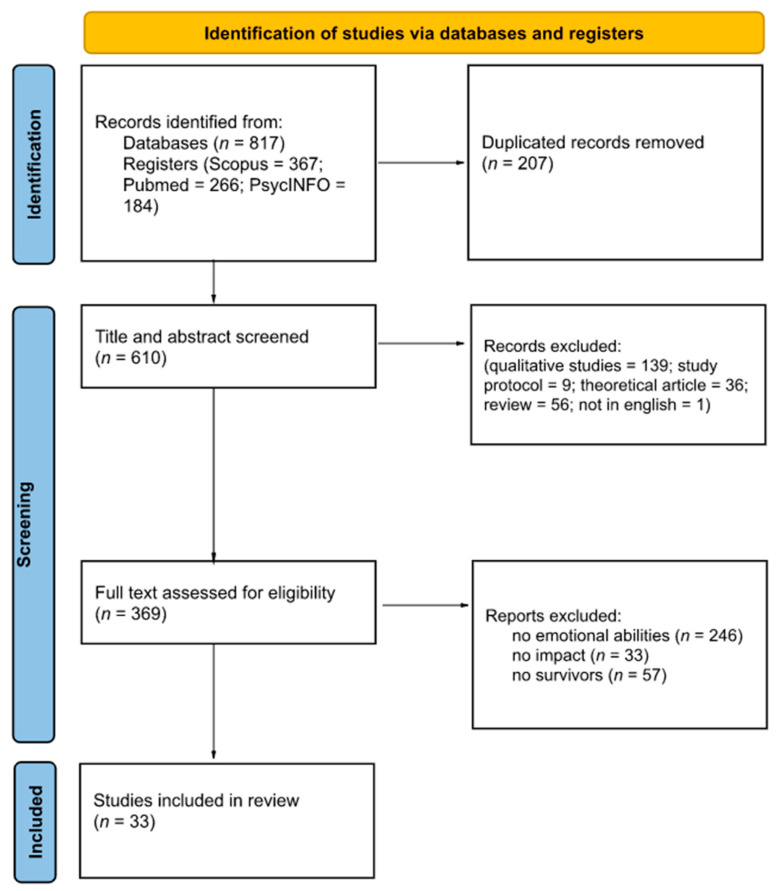
PRISMA diagram.

We conducted a systematic review of the published literature in January 2022 on three databases (PUBMED, PsycINFO, and Scopus) without temporal limits. The present literature review was performed according to PRISMA guidelines [37] (Figure 1 features the systematic review flow), and studies were identified through the keywords “*emotional intelligence*” OR “*emotion regulation*” OR “*mood repair*” OR “*emotional management*” OR “*coping*” AND “*breast cancer survivors*” OR “*breast cancer survivorship*”. These key terms were considered able to retrieve contributions that assess the role of emotion-related abilities in the quality of life of breast cancer survivors. Studies were included if they met the following four criteria: (1) studies that assess emotion-related abilities; (2) studies that examined the impact of emotion abilities on quality of life; (3) breast cancer survivors as a sample (at least one group in case of multiple experimental groups); and (4) studies written in English. As stated in other studies (e.g., [38,39,40,41]), the authors placed a *priori* restrictions by excluding “gray literature” (e.g., doctoral dissertation, conference abstracts, and other non-peer-reviewed sources) to improve review manageability. We placed no limitations on the age of participants and statistical presentation of results.

Moreover, as the keywords show, we did not focus on specific measures of quality of life; indeed, the definition of quality of life is still challenged to this day [42], and it notably varies across different studies, cultures, and methodological approaches. For this reason, we avoided looking only for studies that explicitly mentioned the quality of life; on the contrary, we demanded the quality of life features to the further selection phases, therefore including any study that analyzed the effects of emotional abilities on variables relevant to the quality of life (e.g., psychological well-being, resilience, psychological distress, adjustment to the disease, reduction in psychopathological symptoms, etc.). This allowed the search to identify useful studies beyond the limitations inherent to the concept of the “quality of life”, and to recognize the importance of research focused on specific variables that affect the overall quality of breast cancer survivors’ everyday living and health management.

### Study Selection

The search process resulted in the identification of 817 articles. We then removed 207 articles as duplicates. The first screening was done on the title and abstract of the resulting 610 contributions. Four researchers (I.D., S.T., L.S., and V.S.) coded the studies. Discrepancies were resolved through discussions between raters to reach a consensus.

Only research articles have been considered (reviews, opinions, study protocol, and editorials were excluded). According to Marzorati and colleagues [43], we considered breast cancer survivors all the participants who have completed the oncological treatments and satisfied the defined inclusion criteria.

At the end of this first screening phase, 241 articles were excluded. Subsequently, the full texts of the retrieved articles (369) were analyzed to identify articles that involved emotional abilities to improve the quality of life in breast cancer survivors. We excluded articles in which the term “coping” or others relevant for the present review were used in a broad sense (e.g., patients engaging in activities and hobbies to improve wellness but not in the sense of emotion regulation strategies). Secondarily, we also excluded articles that used the term “breast cancer survivors” but actually featured other samples in the studies (e.g., patients still undergoing treatment; survivors’ caregivers) and articles that dealt with emotion-related abilities to some extent but did not measure their impact on quality of life (e.g., coping abilities were the dependent variable). At the end of this screening phase, 336 articles were excluded. Thus, 33 studies were included in this systematic review (see Figure 1). All the studies were published between 2000 and 2021.

To control for potential selection bias, the raters independently screened 20% of the 33 articles potentially relevant for inclusion in this systematic review. Cohen’s k for the interrater agreement was 1.00.

For each selected study, two researchers extracted in a blinded manner: authors, study design, sample, brief study description, and outcomes of interest for the present review.

## 3. Results

The methodological quality of each study was assessed by two authors independently (L.S. and V.S.). The Cochrane risk of bias tool, version 2 [44] was used to evaluate each domain and its specific risks, indicating as “low risk”, “some concerns”, and “high risk”. The overall quality of the studies is high if the assessment of all the domains results in low. Discrepancies between raters were resolved through discussions with the first and the second authors (I.D. and S.T.). The results of the analysis are presented in Table 1.

The results of the present review clearly support the hypothesis that emotional abilities play multiple important roles in breast cancer survivors’ quality of life and health management (see Table 2 for a synthesis of the studies and Table 3 for a list of the tools used in the selected articles to assess the quality of life or related variables). As clearly summarized in Table 2, all the outcome variables explored in the selected studies fall into different dimension(s) of quality of life: emotional, cognitive, social, physical, and spiritual.

The emotional area was the most investigated area across the reviewed studies. Studies included in this area mainly explored the coping/emotional management, or the adoption of different cognitive strategies to manage one’s emotions. Certainly, emotional abilities have an important impact on the emotional dimension of quality of life as their direct effect is improving the management of affective experiences. However, the reviewed studies clearly show that emotional abilities influence other dimensions of quality of life, depending on the focus of the individual study. For example, when studies focused on patient-physician relationship or the ability to find social support, emotional abilities such as coping demonstrated to help patients communicate their mental and physical health effectively, which could be construed as improvement in the social dimension of QoL [53,60]. Similarly, improvements in physical quality of life were observed in studies that included treatment outcomes and/or adherence to physical exercise [51,58,65,68,70,72]; conversely, dysfunctional emotional management appeared associated with lower physical QoL as well as with fatigue and lower adherence to exercise. Regarding cognitive QoL, active coping skills and emotional intelligence appear associated with the development of cognitive abilities (e.g., reframing) that help patients to avoid biases and potentially-disruptive representations of their condition (e.g., rumination) [53,59,67]. Moreover, active emotional management predicts the ability to adopt healthy mental representations of the illness experience (e.g., benefit finding, acceptance) [46,61]. Finally, two studies included in the sample referred to spiritual aspects, which can be considered a further area for QoL [47,52]: they report that religious beliefs could inform emotional management strategies. Most of the reviewed studies are consistent with one of the main tenets of coping theories, that is, emotional management strategies may be functional or dysfunctional, leading to opposite effects in terms of quality of life in the specific population of breast cancer survivors.

Optimal, functional strategies typically relate to reappraisal, problem-solving and achieving control over emotional situations and stimuli. The reviewed studies showed that the adoption of active coping strategies and problem-solving techniques predicts factors important for quality of life and health management, such as optimism [45], benefit finding [46], vitality and cancer adjustment [47], finding new possibilities, and promoting appreciation for life [48]; they also directly impact quality of life, well-being, and psychological symptoms, such as depression and anxiety [45, 47,49–57]. Additionally, some authors highlighted that coping skills allow breast cancer survivors to improve their cognitive reframing [53], and to reduce distress and fatigue [58]. From a psychological point of view, coping self-efficacy beliefs, and reassurance-seeking behaviors, were significant predictors of lower fear of recurrence [59].

One reviewed study also revealed the impact of coping on physical-related symptoms (i.e., somatic symptoms [60]). Active coping predicts important factors related to social relationships and the relationships with others [48], and support seeking [61].

Other studies showed that the adoption of passive or avoidant coping strategies could have a negative impact on breast cancer survivors’ quality of life [62,63] influencing psychological distress [64,65], and depression [66]. Additionally, disengagement-oriented coping and brooding (indicator of rumination thinking) partially mediated the relationship between social constraints and adjustment [67]. Negative coping, such as dysfunctional coping, could also affect people’s perception of control over their own clinical plan (e.g., treatment control [68]) and could promote negative emotions such as depression and anxiety [69]. Ambivalence over emotional expression was associated with lower follow-up quality of life beyond the effect of expressive suppression [70].

Although these studies highlighted the positive impact of coping strategies on breast cancer survivors’ quality of life, two studies included in the review highlighted some negative results related to the impact of emotional abilities on people’s well-being. Specifically, Ridner and colleagues [71] highlighted that the involvement in intervention focused on improving well-being and the coping strategies resulted in no significant differences between the experimental and control groups. In addition, Lelorain and colleagues [72] reported a non-significant negative relationship between active coping and quality of life and Lyons and colleagues [73] found that coping styles were not correlated with changes in quality of life, depression, or anxiety.

Additionally, emotional intelligence and its sub-components may promote resilience in breast cancer survivors, decreasing vulnerability and mood repair [30]. As highlighted by Rocio Guil and colleagues [30], personal abilities to recognize, discriminate, and regulate emotional states are positively associated with personal growth after the oncological diagnosis. Although the cancer diagnosis is related to negative emotional reactions, it seems that people are able to increase their capacity to repair them effectively.

On the other hand, emotional intelligence is not always beneficial. People who pay great attention to their personal emotions can also have harmful consequences. For example, their ability to regulate emotions could be reduced, acting as a non-protective factor for the promotion of quality of life. This could be related to the level of specific abilities in each person.

We feel it is interesting to report that a number of articles (6) emerged in our first search that did not feature data focused on the impact of emotional abilities but were all focused on the specific construct of self-compassion. We discussed the possibility that self-compassion could be considered an emotional ability such as coping or emotional intelligence, but we decided it is not an “ability” in a strict sense. It is more described as a tendency to experience feelings of kindness towards oneself, as well as a compassionate look towards one’s own failures or flaws [77]. In this sense, it seems to be conceptualized more as a personality trait or attitude. Yet, it is possible that it is related to positive reframing of negative events; indeed, it is associated with adaptive coping [77,78] and emotional stability [79] and it contributes to predicting health and well-being also in chronic illness contexts [80,81]. An interesting aim for future review efforts could be to explore the relationship or partial overlapping between emotional abilities and self-compassion.

## 4. Discussion

This review aims to identify the role of breast cancer survivors’ emotional abilities to improve aspects relevant to the overall quality of life, which the studies assessed according to multiple constructs, including coping/emotional management, emotional intelligence, and mood repair. The first relevant aspect emerging from the retrieved articles is that coping/emotional management play multiple important roles in breast cancer survivors’ well-being and health management. In general, the reviewed studies on proactive coping revealed that these strategies positively affect vitality and general adjustment to cancer. Indeed, active coping strategies (e.g., positive reframing and acceptance) affected benefit findings related to cancer experience. However, at least one study [73] reported a non-significant negative relationship between active coping and quality of life, measured by the MOS SF-36 tool. Authors speculated that active coping could become an exhausting strategy during highly stressful events, such as health management after cancer diagnosis. In other words, even active coping strategies may be ineffective and, at the same time, tiring for people dealing with continual health management challenges.

This is consistent with Baziliansky and Coehn’s meta-analysis [36], which was focused on emotion regulation and its effects on psychological distress in cancer survivors: they found that coping strategies were correlated with distress, but also negative and non-significant relationships were reported [82,83]. To sum up, while especially active coping strategies deserve to be promoted in breast cancer survivors, as they appear consistently associated with positive outcomes, clinicians should take into account that the enactment of coping strategies could generate fatigue, especially when individuals have to deal with long-lasting challenges such as health management in chronic conditions.

Taking into consideration all emotional abilities, coping/emotional management was notably over-represented in the reviewed studies.

Much to our surprise, the application of inclusion criteria led to identifying one study only whose main theoretical construct was emotional intelligence [30]. Notably, this study found that EI (higher in breast cancer survivors than in healthy women) predicted resilience. This result supports the idea that emotional intelligence directly affects one’s ability to deal with challenging circumstances, withstanding and adapting to traumatic and adverse events. This calls for further research on the relationship between personal resources in cancer survivorship and emotional intelligence. Even when it is understood as “perceived EI” or “emotional self-efficacy”, this emotional ability goes beyond the tendency to adopt one more or less effective coping strategy and reflects breast cancer survivors’ perceived ability to understand and manage their own and others’ emotions. As speculation, a patient/survivor who has high emotional intelligence is not more able to manage a specific emotion only, but also to effectively design their own journey across care and health management (e.g., by better understanding their doctor; by helping caregivers in their task without feeling like a burden; by engaging in less biased decision making about treatment and changes in lifestyle; etc.). Indeed, emotional intelligence entails access to a rich “toolbox” of resources one could effectively employ to adapt to challenging contexts, this way improving resilience. Accordingly, one recent study [84], published after the completion of this review process, found that perceived EI and survivorship predicted 37.8% of the variance of depression; the article also suggested that subcomponents of EI are particularly important to develop when facing the experience of breast cancer, namely emotional clarity (the extent to which individuals can unambiguously identify, label, and mentally represent the type and source or of emotions they feel) and emotional repair (the capacity to successfully improve negative moods). It seems that emotional intelligence deserves more research in the field of psycho-oncology, paying attention to its impact on quality of life and protection against negative outcomes such as depression. Its conceptualization as a form of emotional self-efficacy or actual intelligence may be more helpful to develop interventions aimed at its improvement than the conceptualization as a personality trait, that hints at stability over time and leaves less room for modification. Furthermore, it could be interesting to analyze by dedicated research how specific emotional intelligence sub-constructs affect specific needs of breast cancer survivors across their health journey (e.g., when accessing health services, when building social support, or others). Regarding the third emotional ability, namely mood repair, the present review did not yield results focused on this construct specifically, besides the study by Rocio-Guil and colleagues [30], which considered it a component of emotional intelligence. Mood repair is associated with the ability to modify one’s own overall mood during the day, as a subtle, unspecific emotional state independent of simulations that could be identified clearly within the environment [35]. As a direction for future research, it could be interesting to explore this specific construct, both in terms of breast cancer survivors’ quality of life and of the actual degree of its independence from the other emotional abilities, which scholars tend to conceptualize as focused on emotions generated by specific stimuli or events.

While the main results of the present review confirm that active coping strategies are important and effective and should be promoted in cancer survivors, future research may explore further the emotion-related abilities, beyond the mere selection of coping strategies. The concept of “emotional management” should not be reduced to one’s ability to reduce or avoid negative emotions. On the contrary, understanding one’s own emotional journey in depth permits the cultivation of new personal resources, as it happens for example in post-traumatic growth [85]. It is possible that new and more nuanced theoretical constructs are needed to guide future studies in the field of emotion-related abilities for chronic health management.

To sum up, this review highlights the relevance of emotional abilities to promote quality of life, well-being and health management in breast cancer survivors. Our study has some limitations. Although three databases can be regarded as a sufficient number for a systematic review, employing more sources could allow future reviews to be more comprehensive on similar issues. Secondarily, we decided not to include “quality of life” as a keyword in order to find contributions relevant to a broad conception of quality of life (even those papers that did not explicitly refer to this construct); while such approach allowed us to include more relevant studies than focused review efforts [36] and possibly to obtain more information on psychological processes related to emotions, it could be interesting to analyze the relationship between emotional abilities and validated measures specifically focused on quality of life. This could be both a direction for future research and reviews, taking into account that the construct of “quality of life” has been recently challenged due to its instability across different theoretical approaches and cultural contexts [42]. Future reviews may also explore other breast cancer survivors’ emotional abilities, aiming at assessing available instruments and proposing tailored psychological interventions. Moreover, the present review highlighted the importance to study the effects of specific emotional abilities on multiple variables relevant to quality of life: aiming for specificity beyond coping strategies would allow health professionals and researchers to design different kinds of interventions to improve breast cancer survivors’ ability to cope with the illness and achieve a desirable level of psychological well-being. It could be interesting to assess emotional management related to specific issues that are common in the experience of chronic patients and survivors, such as for example return to work [86,87], intimacy and couple infertility [88,89], and choice of treatment [90]. Furthermore, the review highlights that the same conceptualization of emotional abilities deserves to be further improved, recognizing their impact within the specific context of individual chronic illnesses and health journeys in order to develop intervention tools focused on patient experience.

## Figures and Tables

**Table 1:** Risk-of-bias analysis of the selected studies.

	Random Sequence Generation	Allocation Concealment	Blinding of Participants and Personnel	Blinding of Outcome Data	Incomplete Outcome Data	Selective Reporting	Other Bias
Guil et al., 2020 [30]	+	-	-	?	?	+	-
Karademas et al., 2007 [45]	-	-	-	?	?	+	-
Wen et al., 2017 [46]	-	-	?	?	-	-	-
Low et al., 2006 [47]	-	-	-	?	-	-	-
Bellizzi et al., 2006 [48]	+	+	+	?	+	+	-
Cheng et al., 2019 [49]	-	-	-	?	-	+	-
Boehmer et al., 2013 [50]	-	-	-	?	-	-	-
Lu et al., 2018 [51]	+	+	+	?	-	-	-
Gall, 2000 [52]	-	-	-	?	-	-	-
Mishel et al., 2005 [53]	+	+	-	?	-	+	-
Johns et al., 2020 [54]	+	?	-	?	?	+	-
Chu et al., 2019 [55]	-	-	-	?	-	+	-
Carpenter et al., 2014 [56]	+	?	-	?	-	+	-
Beatty et al., 2010 [57]	+	?	-	?	-	+	+
Levkovich et al., 2018 [58]	-	-	-	?	-	+	-
McGinty et al., 2015 [59]	+	-	?	?	-	-	-
Karademas et al., 2007 [60]	-	-	-	?	-	-	-
Fischer et al., 2013 [61]	-	-	-	?	-	-	+
Achimas-Cadariu et al., 2015 [62]	-	+	-	?	-	-	-
Charlier et al., 2012 [63]	-	-	-	?	-	-	-
Cohee et al., 2021 [64]	-	-	-	?	?	+	-
Perez-Tejada et al., 2019 [65]	-	-	-	?	?	+	-
Radin et al., 2021 [66]	-	-	-	?	-	-	-
Kolokotroni et al., 2018 [67]	-	-	-	?	?	+	-
Lan et al., 2018 [68]	-	-	-	?	-	+	-
Romeo et al., 2019 [69]	-	-	-	?	?	+	-
Lu et al., 2018 [70]	-	-	-	?	-	-	+
Ridner et al., 2020 [71]	+	-	-	?	?	+	-
Lelorain et al., 2011 [72]	-	-	?	?	-	-	-
Lyons et al., 2015 [73]	?	?	-	?	-	-	+
Wonghongkul, et al., 2006 [74]	-	-	-	?	?	+	-
Raque-Bogdan, 2016 [75]	-	-	-	?	-	-	-
Arambasic et al., 2018 [76]	-	-	-	?	-	-	-

*Note.* “+” = low risk of bias; “?” = unclear risk of bias; “-” = high-risk of bias.

**Table 2:** Synthesis of studies included in the review according to study design, sample, aim, outcomes of interest. The “Quality of life area” column refers to the dimension(s) of quality of life that are affected by the study, taking into consideration the outcome variables.

Author	Study Design	Sample	Study Aim	Outcomes of Interest	Quality of Life Area
Guil et al., 2020 [30]	Cross sectional research	167 breast cancer survivors	Correlational study to find the specific processes through which the dimensions of Perceived Emotional Intelligence (PEI) (Emotional Attention, Emotional Clarity, and Mood Repair) can act as a risk or protective factor in the development of resilience	Breast cancer survival and PEI predicted 28% of the variance of resilience. The direct effects showed that emotional clarity and mood repair increased resilience levels; emotional attention played a role in vulnerability, decreasing mood repair, and resilience	Emotional
Karademas et al., 2007 [45]	Cross-sectional study	92 breast cancer survivors who had undergone mastectomy	Path analysis on the predictive relationships between self-efficacy, coping, stress, time since diagnosis and since mastectomy, and optimism	Illness-related stress exerted influence on optimism through coping, whereas self-efficacy exerted influence both directly and through coping. Both main coping strategies predicted optimism (positive reappraisal positively and behavioral avoidance negatively)	Emotional
Wen et al., 2017 [46]	Cross sectional study	148 breast cancer survivors	To investigate the extent to which coping strategies, psychosocial distress (perceived stress and depression), and social support were associated with benefit finding	Active coping and depressive symptoms accounted for 20% of the variance in benefit finding	Emotional, Cognitive
Low et al., 2006 [47]	Longitudinal design	558 breast cancer survivors	- To examine emotional approach coping (EAC) strategies and other coping processes as predictors of adjustment over time in women who had recently completed medical treatment for breast cancer - To explore the effects of contextual stressful life events on adjustment over time To examine whether the context in which cancer occurs might influence the predictive value of coping processes on distress	- EAC and other approach-oriented strategies are associated with better general and cancer-specific adjustment, whereas avoidance-oriented coping (i.e., denial) is associated with adverse psychosocial outcome - Lower contextual life stress and greater use of EAC were each associated with greater vitality at baseline Greater EAC was significantly associated with lower CES–D scores at baseline - Greater use of EAC was related to higher PTGI scores as was Positive Reframing, Religious coping, and Problem-Focused coping - In the context of low life stress, the use of more EAC predicted an increase in vitality at 6-months, whereas lower EAC predicted lower vitality - At 12-months, significant cancer-specific EAC was significantly associated with vitality among women who had experienced lower levels of contextual life stress - Greater use of cancer-specific EAC at baseline was associated with a decrease in depressive symptoms, whereas lower EAC scores predicted more depressive symptoms - In the context of higher life stress, this effect was reversed. Greater denial was significantly associated with an increase in depressive symptoms at 6-months - At 12-months, EAC predicted a decrease in depressive symptoms when women had experienced relatively low levels of stressful life events - At 12-months, greater use of cancer-specific denial coping at baseline predicted more cancer-specific distress	Emotional, Social, Spiritual
Bellizzi et al., 2006 [48]	Cross sectional study	224 breast cancer survivors	To examine contextual, disease-related, and intraindividual predictors of posttraumatic growth	Age at diagnosis, marital status, employment, education, perceived intensity of disease, and active coping accounted for 34%, 35%, and 28% of the variance in growth in relationships with others, new possibilities, and appreciation for life.	Social, Emotional
Cheng et al., 2019 [49]	A three-wave longitudinal study	248 breast cancer survivors	Participants completed a package of psychological inventories to evaluate cancer coping style, psychological distress, anxiety and depression, and quality of life	Two cancer-coping classes were identified through LPA, namely adaptive and maladaptive cancer coping. The identified cancer-coping styles predicted survivors′ psychological symptoms, psychological well-being, and health-related quality of life	Emotional
Boehmer et al., 2013 [50]	Cross sectional study	180 lesbian and bisexual breast cancer survivors	To determine differences between lesbian and bisexual cancer survivors to examine whether sexual minority–specific issues contribute to these survivors′ adjustment	Preoccupation coping was associated with worse mental health, more social support, more fatalism, or fighting spirit coping and better future perspective was associated with lower depression. Hopelessness coping was associated with more depression symptoms. Fighting spirit coping and better future perspective related to less anxiety	Emotional
Lu et al., 2018 [51]	Randomized controlled trial with three arms	136 breast cancer survivors	To examine the impact of expressive writing on quality of life	The enhanced self-regulation condition had a large and statistically significant effect, and the self-regulation condition had a small effect on quality-of-life improvement compared with the cancer-fact group	Emotional, Social, Cognitive, Physical
Gall, 2000 [52]	Cross sectional study	52 breast cancer survivors	To explore the role of religious resources in long-term adjustment to breast cancer	Various experiences of relationship with God (e.g., presence) were related to more positive appraisals of the current cancer situation as well as to the greater use of the nonreligious coping behavior of focusing on the positive. The same coping behavior, for example religious avoidance, could be related to both positive and negative appraisals of the cancer situation. Religious resources, but not nonreligious resources predicted emotional and spiritual well-being for long-term breast cancer survivors	Emotional, Spiritual
Mishel et al., 2005 [53]	Randomized controlled trial	509 breast cancer survivors (360 Caucasian, 149 African–American women)	To test the efficacy of a “uncertainty management” intervention, focused on augmenting the usage of active vs. passive coping strategies	Training in active coping skills resulted in improvements in cognitive reframing, cancer knowledge, patient–health care provider communication, and coping skills	Cognitive, Social
Johns et al., 2020 [54]	Evidence-based interventions	91 breast cancer survivors	Intervention to examine the feasibility and preliminary efficacy of group-based acceptance commitment therapy (ACT, focused on coping strategies) for fear of recurrence and quality of life, compared with survivor education and usual care	All interventions improve fear of recurrence and quality of life but ACT obtained better results in the same constructs than both survivor education and usual care	Emotional
Chu et al., 2019 [55]	Experimental study	96 breast cancer survivors	Participants were involved in expressive writing, three groups: writing about stress coping and finding benefits vs. emotional disclosure vs. objective cancer facts	Coping and cancer facts writing groups had fewer PTSD symptoms than emotional disclosure group	Emotional
Carpenter et al., 2014 [56]	Randomized waitlist-controlled trial	132 breast cancer survivors	To develop an online cognitive behavioral stress management intervention for early-stage breast cancer survivors and evaluate its effectiveness	Higher self-efficacy for coping with cancer and for regulating negative mood and lower levels of cancer-related post-traumatic symptoms were found in the experimental group	Emotional
Beatty et al., 2010 [57]	Randomized controlled trial; intervention and control group tested at baseline and at 3 and 6 months after	40 breast cancer survivors	To test the effect of an intervention based on a self-help workbook for improving adjustment for breast cancer survivors	Control participants used less venting coping than workbook ones. Reliable change indices showed a trend towards a protective effect across all coping measures for workbook participants	Emotional
Levkovich et al., 2018 [58]	Cross sectional study	170 breast cancer survivors, stages I–III, 1–12 months post-chemotherapy	- To examine the nature of the symptom cluster of emotional distress, fatigue, and cognitive difficulties. (BCS); - To assess the mediating role of subjective stress and coping strategies (emotional control and meaning-focused coping) in the association between age and symptom cluster	Emotional control was negatively associated with distress and meaning-focused coping was negatively associated with distress and fatigue	Emotional, Physical
McGinty et al., 2015 [59]	Longitudinal study	161 breast cancer survivors	To assess and predict fear of cancer recurrence during a critical event in cancer survivorship	Cognitive Behavioral Model variables, including risk, severity, coping self-efficacy beliefs, and reassurance-seeking behaviors, were significant predictors of lower fear of recurrence	Emotional, Cognitive
Karademas et al., 2007 [60]	Cross sectional study	103 Greek breast cancer survivors and 100 comparison group	To investigate the association of cancer-related stress and coping with psychological health (and especially with those aspects of psychological health exhibiting a significant difference between breast cancer survivors and healthy controls)	Cancer-related stress and coping explained an additional 26% of the somatic symptom variance, 25% of the anxiety variance, 24% of the social dysfunction variance, as well as 29% of the depression variance. They also explained an additional 32% of the overall GHQ score variance. Depressive symptoms were positively predicted by stress and behavioural avoidance, and negatively by the use of social support. Behavioural avoidance was positively predicted by stress	Social, Emotional
Fischer et al., 2013 [61]	Cross sectional and longitudinal study	57 breast cancer survivors	- To analyze to what degree illness perceptions and coping are associated with psychological distress in women who wish to participate in a psycho-educational group intervention for breast cancer survivor -To examine how participants′ illness perceptions, coping style, and distress change after participating in the intervention. To investigate to what extent distress at follow-up is related to baseline values and changes in illness perceptions and coping style	- Distress was positively related to beliefs about the consequences of breast cancer, chronic timeline, cyclical timeline, and emotional representations. An inverse association was observed between distress and illness coherence - Problem-focused coping was related to higher scores for support seeking/venting of emotions and acceptance - Greater use of avoidance as a coping strategy was strongly related to higher distress scores, whereas acceptance was inversely related to distress - A linear trend was observed for social support seeking/venting of emotions for which mean scores declined steadily over time. Avoidance and acceptance showed a quadratic trend in that they were used more often directly after the programme, but less frequently after 1 year. Problem-focused coping scores did not change between the three assessment points - Greater use of avoidance at baseline was associated with higher distress at T2. Interestingly, whereas the use of acceptance as coping strategy at baseline was related to lower distress 1-year after start of the course (T3), an increase in the use of acceptance over time (change score) was related to greater distress at T3	Emotional, Cognitive
Achimas-Cadariu et al., 2015 [62]	Cross sectional study	51 breast cancer survivors and 59 control group	- To compare multidimensional constructs of quality of life, emotional distress, anxiety, and cognitive coping status of women with premalignant and malignant breast disease during the survival stage and healthy control group; - To identify the potential effect of breast cancer and psychosocial predictors on quality of life, effects adjusted for covariates	Statistically significant negative effect of emotional distress and of the catastrophizing coping strategy on quality of life	Emotional, Social, Cognitive, Physical
Charlier et al., 2012 [63]	Cross sectional study	440 breast cancer survivors	To cluster cancer survivors according to their symptoms and psychosocial variables with the aim to identify survivors with a homogenous psychosocial profile. To look for differences in physical activity level and supportive care needs for physical activity among the resulting clusters	- Women in cluster 1 (low distress-active approach) were using more problem-oriented coping - Women in cluster 4 (high distress-emotional approach) used emotional coping more than the other clusters - Women in cluster 2 (low distress-resigned approach) reported significant lower levels of problem-oriented and avoidance coping, but were using significant less emotional coping - Women from cluster 3 (high distress-active approach) were frequently using problem-oriented and avoidance coping strategies -Women in cluster 1 and 2 had significantly less quality-of-life issues than the other two clusters in several areas such as fatigue, body image, self-esteem, and personal and treatment control	Cognitive, Emotional
Cohee et al., 2021 [64]	Cross sectional study	1127 breast cancer survivors who were 3 to 8 years post-diagnosis	Multiple mediation analyses were conducted to determine whether avoidant coping mediated the relationship between each distress variable and each well-being variable	Avoidant coping significantly mediated the relationship between each well-being variable and each distress indicator. Avoidant coping mediated 19–54% of the effects of the contributing factors on the distress variables	Emotional
Perez-Tejada et al., 2019 [65]	Cross-sectional descriptive design	54 breast cancer survivors	Pilot study to determine whether different coping strategies are associated with differences in psychological distress, cortisol, and tumor necrosis factor alpha (TNF-a) levels in breast cancer survivors	Passive coping strategies were associated with higher psychological distress, cortisol, and TNF-a levels	Physical, Emotional
Radin et al., 2021 [66]	Cross sectional study	171 breast cancer survivors	To examine correlations between executive functions (EF), coping, and depressive symptoms in breast cancer survivors. To longitudinally test the hypothesis that coping mediates the relationship between EF and depressive symptoms	EFs were correlated with avoidant coping. In longitudinal analyses, use of the avoidant strategy behavioral disengagement at 1-year mediated the association between objective and subjective EFs at 6 months and depressive symptoms at 2 years	Emotional
Kolokotroni et al., 2018 [67]	Cross sectional study	125 breast cancer survivors	Investigated the mediating psychological pathways through which social constraints on cancer-related disclosure, low optimism, disengagement-oriented coping, and brooding could be associated with low levels of psychosocial adjustment	Disengagement-oriented coping and brooding (indicator of rumination thinking), partially mediated the relationship between social constraints and adjustment	Emotional, Cognitive, Social
Lan et al., 2018 [68]	Cross-sectional study	124 breast cancer survivors	Survey to assess the relationship between illness perception, coping style, functional exercise adherence, and demographic and illness-related characteristics	Dysfunctional coping strategies were negatively associated with treatment control	Physical (adherence to treatment and exercise)
Romeo et al., 2019 [69]	Cross sectional study	123 breast cancer survivors	To analyze both positive and negative outcomes after cancer diagnosis, through an extensive analysis of different potentially relating factors, which can be deeply associated with the patients′ ability to manage the disease	“Fatalism” coping strategy and perceived social support were two significant predictors of post traumatic growth. The “Helpless-Hopeless” and “Anxious Preoccupation” coping strategies, as well as an insecure attachment style, were significant predictors of depression, while the “Anxious Preoccupation” coping strategy and an insecure attachment style were significant predictors of anxiety	Emotional
Lu et al., 2018 [70]	Cross sectional study	103 breast cancer survivors	To examine the longitudinal effects of expressive suppression, ambivalence over emotional expression (i.e., inner conflict over emotional expression), and cognitive reappraisal on quality of life	Ambivalence over emotional expression was associated with lower follow-up quality of life above and beyond the effect of expressive suppression. Cognitive reappraisal moderated the relations between expressive suppression and follow-up quality of life	Emotional, Social, Cognitive, Physical
Ridner et al., 2020 [71]	Experimental study	160 breast cancer survivors with lymphedema	To compare a web-multimedia intervention that included information on coping strategies with an informational pamphlet to improve well-being	No significant differences between the groups; the role of coping strategies is unclear as they were one of multiple contents of the web-based intervention	/
Lelorain et al., 2011 [72]	Cross sectional study	298 breast cancer survivors and 132 comparison group	To explore this issue by comparing quality-of-life prediction between cancer survivors and a comparison group	-Substance abuse and active coping lead to decreased quality of life - Although not significant, a negative relation between active coping and mental quality of life is reported; authors speculate that active coping may sometimes be exhausting	Emotional, Social, Cognitive, Physical
Lyons et al., 2015 [73]	Two experimental studies	31 breast cancer survivors	To develop and pilot test an intervention to optimize functional recovery for breast cancer survivors	Reductions in self-blame were associated with reductions in depression. The change scores for the other three coping styles were not correlated with changes in quality of life, depression, or anxiety	Emotional
Wonghongkul, et al., 2006 [74]	Cross sectional study	150 breast cancer survivors	-To explore the levels of uncertainty in illness, types of stress appraisal, types of coping, and levels of quality of life in breast cancer survivors - To examine predictors of quality of life in breast cancer survivors including uncertainty in illness, stress appraisal, and coping	Distancing coping predict quality of life; seeking social support reduces stress among breast cancer survivors	Social, Emotional
Raque-Bogdan, 2016 [75]	Cross sectional study	275 breast cancer survivors	To test a model of well-being recovery. Structural equation modeling was used to examine relationships between affect, loneliness, self-compassion, self-efficacy for coping with cancer, well-being, and life satisfaction	Coping efficacy was a consistent mediator in the path sequences from positive affect, negative affect, and loneliness to emotional well-being and life satisfaction	Emotional
Arambasic et al., 2018 [76]	Cross sectional research	82 breast cancer survivors	To extend the association between attachment styles and psychological adjustment to the context of long-term breast cancer survivors and to determine whether lower self-compassion underlies this association	Higher attachment anxiety and attachment avoidance were significantly and positively associated with stress and perceived negative impact of cancer. Significant indirect effects of attachment anxiety and attachment avoidance (on both stress and perceived negative impact of cancer) through lower self-compassion	Emotional

**Table 3:** List of the tools used to assess Quality of Life or related variables and related ranges.

Author	Quality of Life or Related Variables′ Tools
Guil et al., 2020 [30]	- Wagnild and Young Resilience Scale (range = 25–175; Non-resilience (25–74); Low resilience (75–100); Average resilience (101–125); High resilience (126–150), and Very high resilience (151–175)
Karademas et al., 2007 [45]	- Personal Optimism Scale from the Questionnaire for the Assessment of Personal Optimism and Social Optimism-Extended, range = 8–32 with higher scores indicating higher optimism
Wen et al., 2017 [46]	- The Perceived Stress Scale (range = 0–56, with higher scores indicating greater overall stress) - A scale for benefit finding with range = 1–5 and higher score indicating higher benefit finding - The Patient Health Questionnaire (PHQ), score 0–27 with higher score indicating higher depression
Low et al., 2006 [47]	- Vitality subscale from the Medical Outcomes Study Short Form (SF–36). range = 0–100 with higher scores indicating lower vitality issues - The Center for Epidemiologic Studies–Depression Scale (CES–D), range = 0–88 with higher scores indicating higher depression - The Revised Impact of Event Scale (IES–R), range = 0–88 with higher scores indicating more distressing cancer-specific intrusive thoughts, avoidance, and hyperarousal - Post-Traumatic Growth Inventory (PTGI), (range = 0–105, with high scores indicating positive growth)
Bellizzi et al., 2006 [48]	- Post-traumatic Growth Inventory (range = 0–105, with high scores indicating positive growth)
Cheng et al., 2019 [49]	- Distress Thermometer - Hospital Anxiety and Depression Scale (range = 0–21, with a score of 8 or more suggesting a clinically significant level of depression/anxiety symptoms) - 36-Item Short Form Survey (Health) (range = 0–10, with higher score indicating better health)
Boehmer et al., 2013 [50]	The European Organization for Research and Treatment of Cancer, Quality of Life Questionnaire-Breast Cancer (EORTC QoL-BR23) (range = 0–100; a high score for functional scales and for Global Health Status/QoL represent better functioning ability or HRQoL, whereas a high score for symptom scales and single items represents significant symptomatology)
Lu et al., 2018 [51]	- Functional Assessment of Cancer Therapy general scale (FACT-G), range = 0–108 with higher scores indicating higher quality of life
Gall, 2000 [52]	- Spiritual Well-Being Scale (SWBS) (ranges = 20–120, with a higher score representing greater spiritual well-being) - Brief Symptom Inventory (BSI) (range = 0–212 as a global index was used, with higher scores indicating higher psychological distress) - Life Satisfaction Questionnaire (LSQ) (unclear source, but an average score of 9 items)
Mishel et al., 2005 [53]	- Self-control schedule (two subscales used both with range = 10–100 with higher scores indicating higher cognitive reframing and problems solving, respectively) - Patient/Provider Communication Scale (range = 5–25, with higher scores indicating a greater degree of communication)
Johns et al., 2020 [54]	- Patient-Reported Outcomes Measurement Information System (PROMIS) Global Health Scale—eight items were used, two subscales both range = 4–20 with higher scores indicating higher physical and mental health, respectively - Fear of Cancer Recurrence Inventory–Short Form (range = 0–36, with higher scores indicating greater fear of cancer recurrence)
Chu et al., 2019 [55]	- Symptom Scale—Self report (range = 0–51, with high score indicating more severe symptoms)
Carpenter et al., 2014 [56]	- Cancer Behavior Inventory v2.0 (range = 33–297, higher score indicates more confidence the patient had in his or her ability to perform a specific behavior related to coping with cancer now or some time in the near future’) - Functional Assessment of Cancer Therapy-Breast (range = 0–28, higher score indicates better social and functional well-being) - The Positive and Negative Affect Schedule (PANAS), range of both scales = 10–50 with higher scores indicating more positive affect for the first scale and more negative affect for the second scale - The Revised Impact of Event Scale (IES–R) (range = 0–88, with higher scores indicating more distressing cancer-specific intrusive thoughts, avoidance, and hyperarousal) - Benefit Finding Scale (range = 17–85, with higher score indicating a higher degree of benefit finding)
Beatty et al., 2010 [57]	- Posttraumatic Stress Scale-Self Report (range = 0–51, with higher scores indicating better functioning - European Organization for Research and Treatment of Cancer Quality of Life Core Questionnaire (range = 0–100; a high score for functional scales and for Global Health Status/QoL represents better functioning ability or HRQoL, whereas a high score for symptom scales and single items represents significant symptomatology)
Levkovich et al., 2018 [58]	- Subjective Stress Scale (range = 0–10, with higher score indicating higher stress) - Fatigue Symptom Inventory (range = 0–140, higher scores indicate a higher level of fatigue) - Brief Symptom Inventory—two scales were used, both with range = 0–24 with higher scores indicating higher anxiety and depression respectively - EORTC quality of life questionnaire (range = 0–100; with a high score for functional scales and for Global Health Status/QoL represents better functioning ability or HRQoL, whereas a high score for symptom scales and single items represents significant symptomatology)
McGinty et al., 2015 [59]	- Consequences subscale of the Revised Illness Perception Questionnaire (range = 6–30 with higher score meaning more serious expected consequences of the illness) - Brief Cancer Behavior Inventory (range = 9–126 with higher score meaning higher ability to cope with cancer)- Behavior subscale of the Health Anxiety Questionnaire (range = 4–12 with higher score meaning higher reassurance-seeking behavior) - Visual analogue scale (VASs) - The CancerWorry Scale (CWS) (range = 8–32, higher scores indicate more frequent worries about cancer)
Karademas et al., 2007 [60]	- Personal Optimism Scale from the Questionnaire for the Assessment of Personal Optimism and Social Optimism-Extended, range = 8–32 with higher scores indicating higher optimism - Resilience Self-efficacy Scale (range = 7–28 with higher scores indicating higher resilience self-efficacy)
Fischer et al., 2013 [61]	- The 25-item Hopkins Symptom Check List (HSCL-25), range 1–4 with higher scores indicating higher distress related to one’s illness and a cut-off of 1.75 indicating clinically relevant distress (in the reviewed paper, the authors used sum of the items and a cut-off of 39 for “elevated distress”) - The Illness Perception Questionnaire-Revised (IPQ-R) (eight subscales used)—all subscales have range = 1–5 besides the Illness identity subscale that has range = 0–14. Higher scores indicate stronger perception of specific aspects of one’s illness, e.g., self-efficacy to manage it, variability and predictability of symptoms, negative emotions, etc.
Achimas-Cadariu et al., 2015 [62]	- Beck Depression Inventory-Second Edition (BDI-II) (range = 0–63, with higher score indicating severe depression) - The Endler Multidimensional Anxiety Scales (EMAS), composed by three scales each composed by other subscales; EMAS-S for state anxiety and EMAS-T for trait anxiety (range of any subscale = 1–75 with higher scores indicating lower anxiety) and EMAS-P for anxiety towards specific threats (ranges = 0–5 with higher scores indicating higher anxiety) - Functional Assessment of Cancer Therapy-Breast (FACT-B) (range = 0–164, with higher scores indicating better quality of life).
Charlier et al., 2012 [63]	- The European Organization for Research and Treatment of Cancer, Quality of Life Questionnaire-Breast Cancer (EORTC QoL-BR23) (range = 0–100; a high score for functional scales and for Global Health Status/QoL represent better functioning ability or HRQoL, whereas a high score for symptom scales and single items represents significant symptomatology) - Functional Assessment of Cancer Therapy—Fatigue questionnaire, range = 13–65 with higher score indicating increased fatigue - Hospital Anxiety and Depression Scale (HADS), (range = 0–21, with a score of 8 or more suggesting a clinically significant level of depression/anxiety symptoms - The Illness Perception Questionnaire-Revised (IPQ-R); all subscales have range = 1–5 besides the Illness identity subscale that has range = 0–14. Higher scores indicate stronger perception of specific aspects of one’s illness, e.g., self-efficacy to manage it, variability and predictability of symptoms, negative emotions, etc.
Cohee et al., 2021 [64]	- Center for Epidemiological Studies–Depression scale (range = 0–60, with higher scores indicating more serious symptoms. A cut-off score of 16 suggests that individuals are at risk for clinical depression)
Perez-Tejada et al., 2019 [65]	- Hospital Anxiety and Depression Scale (HADS) (range = 0–21, with a score of 8 or more suggesting a clinically/significant level of depression/anxiety symptoms) - Cortisol (stress level)
Radin et al., 2021 [66]	- Higher level cognitive complaints subscale of the Patient’s Assessment of Own Functioning Inventory (PAOFI), range = 1–6 with higher scores indicating more complaints related to executive functioning - The Beck Depression Inventory (BDI-II), with the subsequent cut offs: 0–13, mini- mal depression; 14–19, mild depression; 20–28, moderate depression; and 29–63, severe depression
Kolokotroni et al., 2018 [67]	-.Psychosocial Adjustment to Illness Scale–Self-Report, a total score was computed with -range = 0–100 with higher scores indicating higher psychosocial adjustment - Social Constraints Scale (range = 15–60, where the higher the score, the more the social constraints)
Lan et al., 2018 [68]	- Functional Exercise Adherence Scale (FEAS) for Postoperative Breast Cancer Survivors composed by three subscales: “adherence to physical exercise”, range = 9–45; “adherence to seeking advice”, range = 4–20; “adherence to following precautions”, range 5–25, all with higher scores indicating higher adherence - The Illness Perception Questionnaire-Revised (IPQ-R); all subscales have range = 1–5 besides the Illness identity subscale that has range = 0–14. Higher scores indicate stronger perception of specific aspects of one’s illness, e.g., self-efficacy to manage it, variability and predictability of symptoms, negative emotions, etc.
Romeo et al., 2019 [69]	- Post-Traumatic Growth Inventory (range = 0–105, with high scores indicating positive growth) - Hospital Anxiety and Depression Scale (range = 0–21, with a score of 8 or more suggesting a clinically significant level of depression/anxiety symptoms)
Lu et al., 2018 [70]	- Functional Assessment of Cancer Therapy (FACT-G) - Emotional Expressivity Questionnaire (AEQ) (range = 22–154, with higher scores indicating higher tendency to express emotions)
Ridner et al., 2020 [71]	Lymphedema Symptom Intensity and Distress Scale–Arm (LSIDS-A) (range 1–100), in which individual weighted values are subsequently average to reach an overall index of symptom burden Profile of Mood States-Short Form (POMS-SF) (range 0–100); responses are summed to provide a total mood disturbance score Perceived Medical Condition Self-Management Scale (range = 8–40 with higher scores indicating higher health competence) 19-item Medical Outcomes Study Social Support Survey (MOS Social Support Survey) (range 1–95), with higher scores indicating greater levels of social support
Lelorain et al., 2011 [72]	- Visual analogue scale (VASs) - Bruchon-Schweitzer social support questionnaire (source unclear on exact range; 16 items and four subscales with higher scores indicating higher social support; only the total score was used) - Medical Outcome Study Short Form-36 (MOS SF-36) (multiple scales with multiple response types on different areas related to patient reported outcomes; higher scores in a given area indicate higher issues for the patient)
Lyons et al., 2015 [73]	- The Functional Assessment of Cancer Therapy-Breast Cancer + Arm Morbidity (FACT-B+4), (range = 0–164, with higher scores indicating better quality of life). - The Hospital Anxiety and Depression Scale (HADS), (range = 0–21, with a score of 8 or more suggesting a clinically significant level of depression/anxiety symptoms)
Wonghongkul, et al., 2006 [74]	- Stress Appraisal Index composed by three scales all with range 0–10 with higher scores indicating higher appraisal of stress in terms of harm, threat, and challenge, respectively - Quality of Life: Breast Cancer Version Questionnaire (range = 0–460; a higher score indicates higher quality of life)
Raque-Bogdan, 2016 [75]	- The Positive and Negative Affect Schedule (PANAS), range of both scales = 10–50 with higher scores indicating more positive affect for the first scale and more negative affect for the second scale - Cancer Behavior Inventory—Brief Version (CBI-B), range = 9–126 with higher score meaning higher ability to cope with cancer - The emotional well-being subscale of Functional Assessment of Cancer Therapy—Breast Cancer Version (FACT-B), range = 0–20 with higher scores indicating higher emotional well-being - Satisfaction with Life Scale (SWLS), range = 5–35 with higher scores indicating higher satisfaction with life
Arambasic et al., 2018 [76]	- 20-item Negative Impact Summary scale of the Impact of Cancer scale Version 2 (negative IOC), range = 1–5 with higher scores indicating a more negative impact of cancer

## Data Availability

All data generated or analyzed during this study are included in this published article.
